# The Effects of Fungal Pathogen Infestation on Soil Microbial Communities for *Morchella sextelata* Cultivation on the Qinghai–Xizang Plateau

**DOI:** 10.3390/jof11040264

**Published:** 2025-03-28

**Authors:** Ming-Chen Guo, Bo-Chun Wu, Cai-Yun Luo, Wei Sa, Le Wang, Zhong-Hu Li, Qian-Han Shang

**Affiliations:** 1 State Key Laboratory of Plateau Ecology and Agriculture, Qinghai University, Xining 810086, China; 13633436166@163.com (M.-C.G.); wubochunpie@163.com (B.-C.W.); sawei3699@163.com (W.S.); wangleqhu@163.com (L.W.); 2 Key Laboratory of Resource Biology and Biotechnology in Western China, Ministry of Education, College of Life Sciences, Northwest University, Xi’an 710069, China; 202310286@stumail.nwu.edu.cn (C.-Y.L.); lizhonghu@nwu.edu.cn (Z.-H.L.)

**Keywords:** *Morchella*, metagenomic sequencing, soil microbial community, fungal diseases, Qinghai–Xizang Plateau

## Abstract

Fungi infestation as a disease has serious impacts on the cultivation of *Morchella* species. To investigate the effects of fungi infestation on the microbial diversity and community structure of soil when cultivating *Morchella sextelata*, we sampled soil samples of *Morchella* cultivars in the Qinghai–Xizang Platea and used metagenome sequencing technology to identify the disease fungi and analyze the differences in microbial diversity and structure between disease-infested and healthy soils. The disease fungi identified were *Tricharina gilva* and *Peziza lohjaoensis*, and the microbial diversity of *T. gilva*-infected soil was higher than that of healthy soil, while the diversity of *P. lohjaoensis*-infected soil was lower. Interestingly, whether infected with *T. gilva* or *P. lohjaoensis*, the soil microbial community was changed, and the dominant phyla and genera were different in different soil samples. When infected with *P. lohjaoensis*, the dominant phyla with relatively high abundances included Proteobacteria, Bacteroidetes, and Ascomycota, with average relative abundances of 44%, 18%, and 15%, respectively, and the dominant genera with high relative abundances encompassed *Pseudomonadaceae*, *Terfezia*, and *Pedobacter*, with average relative abundances of 8%, 9%, and 5%, respectively. Following infection with *T. gilva*, the dominant phyla with higher relative abundances were Proteobacteria, Acidobacteria, and Bacteroidetes, with average relative abundances of 46%, 15%, and 12%, respectively, and the dominant genera with high relative abundances included *Hydrogenophaga*, *Sphingomonas*, and *Polaromonas*, with average relative abundances of 9%, 3%, and 2%, respectively. Additionally, we found that lipid-metabolism-related genes were less abundant in the soil infected with *P. lohjaoensis* than in the other soil samples, and glycoside hydrolase diversity was lower in the soil infected with *T. gilva* than in other healthy soils. The results showed that the effects of different disease fungi on soil microbial communities and functional genes were different, which provided a theoretical basis for the sustainable cultivation of *Morchella*.

## 1. Introduction

*Morchella,* a genus within the Morchellaceae family of the order Pezizales, subphylum Ascomycota, and class Discomycetes, is a rare group of fungi that holds both edible and medicinal values [1]. Most species of *Morchella* grow in temperate regions of the Eurasian continent and North America in the Northern Hemisphere [2]. Morphologically, the genus can be broadly categorized into three main types: black morels, yellow morels, and half-free morels [3]. Morels are rich in amino acids, polysaccharides, and trace elements [1]. Extensive research has demonstrated that *Morchella* species exhibit a variety of medicinal properties, including antioxidant, antibacterial, hypoglycemic, and immune-enhancing effects [4,5,6]. According to the statistics of the China Edible Fungi Association, China’s domestic production was only 111 tons in 2010 but reached 78,500 tons in 2019, an increase of 191.59% over 2018, and 137,000 tons in 2020, an increase of 89.22% over 2019. The edible fungi industry, including morel mushrooms, adds efficiency to China’s agriculture. It has played an important role in increasing rural income and poverty alleviation [7]. However, in recent years, wild *Morchella* populations have significantly declined due to climate change, habitat degradation, and overharvesting [8]. As a result, the demand for wild *Morchella* resources is no longer met by natural populations, creating a pressing need for alternative sources [9]. The artificial cultivation of *Morchella*, while still in its developmental stages, has made considerable progress. Notably, researchers in the United States succeeded in cultivating *Morchella* fruiting bodies indoors in the 1980s, with the world’s first patent for indoor cultivation granted in 1986 [10,11]. Currently, the strains used in *Morchella* cultivation are mostly derived from domesticated wild *Morchella* species. In China, the main varieties of artificially cultivated *Morchella* include *Morchella importuna*, *M. sextelata*, and *M. septimelata* [12]. Artificial cultivation techniques for *Morchella* generally fall into three main categories: facility-based cultivation, field planting, and woodland cultivation [13,14,15,16]. Despite these advances, the stability of current cultivation methods remains low, with challenges related to fungal contamination and the presence of polluted strains. These factors complicate disease management and control, presenting significant obstacles to the large-scale and sustainable production of *Morchella* [17].

The metagenome, also known as the pangenome, was a concept first proposed by Handelsman et al. [18]. In recent years, some researchers have discovered that metagenome sequencing can provide taxonomic information and functional gene data for microbiota, enabling further analysis of microbial community diversity, population evolutionary relationships, functional activities, and interactions with host environments [19]. For example, Zhang et al. [20] utilized metagenomic sequencing to explore soil properties in maize cultivation, revealing that continuous cropping significantly impacts soil microbial diversity, community structure, and metabolic profiles. Similarly, Andrew et al. [21] applied metagenomics to discover a significant relationship between fungal community differences and the geographic distance of plant populations, highlighting the important roles of genes and the environment in shaping foliar fungal communities. In another study, Xu et al. [22] conducted a study on the microbial composition and functions of newly cultivated farmland, slope farmland, and silt dam on the Loess Plateau based on metagenomics, indicating that microorganisms typically cope with variable environments through functional redundancy. Additionally, Wang et al. [23] demonstrated that metagenomics technology can aid in studying the interactions of microbial communities in plant rhizospheres and elucidating the mechanisms by which key microorganisms affect plant growth. Collectively, these studies underscore the power of metagenomics in deciphering the complex interactions within microbial ecosystems.

The honeycomb-like depressions on the caps of *Morchella* fruiting bodies provide an excellent habitat for pathogenic microorganisms, making *Morchella* highly susceptible to infection by various environmental pathogens, including fungi and bacteria, during its growth stages [24,25,26]. With the expanding cultivation of *Morchella*, disease outbreaks have become more frequent, prompting increasing attention to be paid to research on *Morchella* pathogens. For instance, Guo et al. [25] identified *Fusarium incarnatum* as the causative agent of stem rot in *Morchella*, while Lv et al. [26] reported *Lecanicillium aphanocladii* as the pathogen responsible for decay in *Morchella*. Additionally, Yu et al. [27] found *Aspergillus* species to be the causative agents of white rot in *Morchella*, and Zhang et al. [28] demonstrated that *Rhizopus stolonifer* can also infect *Morchella*. Liu’s research revealed that *Fuarium nematophilum* is responsible for stem rot in *Morchella sextelata* [29], while Gao Zhanghui et al. [30] showed that *Staphylococcus heterophyllum* causes cobweb disease in *Morchella*. However, to date, few researchers have studied the microbial communities in the soil where *Morchella* grows. Despite these advances in identifying pathogens associated with *Morchella* diseases, there remains a significant gap in understanding the microbial communities present in the soils where *Morchella* is cultivated. Further research on soil microbiomes and their interactions with *Morchella* could provide critical insights into the dynamics of disease development and help improve cultivation practices.

The composition and diversity of soil microorganisms play a crucial role in the growth and development of fungi. For example, Zhang et al. [31] investigated the rhizosphere soil microorganisms of wild *Morchella* in Yili and found that soils associated with *Morchella* exhibited significantly higher species richness compared to non-*Morchella* soils. In a study by Chen et al. [32], the microbial community of *Morchella* affected by stem rot disease was analyzed using Illumina MiSeq high-throughput sequencing. Their results revealed a notable shift in the dominant fungal communities in the affected soils. Similarly, Tan et al. [33] investigated *Morchella* soils infected with Paecilomyces lanosus and observed an increase in the abundance of fungi such as Paecilomyces, Acremonium, and Corynespora in diseased soils compared to healthy controls. These findings highlight the significant influence of soil microbial communities on *Morchella* growth, particularly in the context of fungal diseases. Soil not only serves as a medium for *Morchella* growth but also acts as a habitat for various microorganisms, many of which function as decomposers in the soil food web. Such microorganisms can impact *Morchella* growth in different ways. Xiong et al. [34] demonstrated that a richer bacterial community could promote the development of *Morchella* fruiting bodies, suggesting a positive relationship between microbial diversity and fungal productivity. Additionally, Wang et al. [35] further confirmed that the abundance of bacteria in soils hosting *Morchella* was significantly higher than in soils devoid of the fungus, underscoring the symbiotic relationship between *Morchella* and its soil microbiota. However, few studies have focused on the impact of fungal disease infection on the structure and function of soil microbial communities in *Morchella* cultivation soil in the Qinghai–Xizang Plateau region. This study utilizes metagenomics technology to explore the impact of soil microorganisms on *Morchella* growth and analyzes the differences in soil microbial communities between healthy and fungus-infected conditions, aiming to provide theoretical support for exploring the disease mechanism of *Morchella* and improving artificial cultivation techniques for *Morchella*. Despite these insights, limited research has been conducted on the impact of fungal diseases on the structure and function of soil microbial communities in *Morchella* cultivation, particularly in the unique environmental context of the Qinghai–Xizang Plateau. This study employs metagenomics technology to explore how soil microbial communities influence *Morchella* growth and to analyze the differences in microbial structures between healthy and fungal disease-infected soils. The aim is to provide a deeper understanding of the mechanisms underlying *Morchella* diseases and to offer theoretical support for enhancing artificial cultivation practices of this economically significant fungus in challenging ecological environments.

## 2. Materials and Methods

### 2.1. Soil Sample Collection

**Figure 1:**
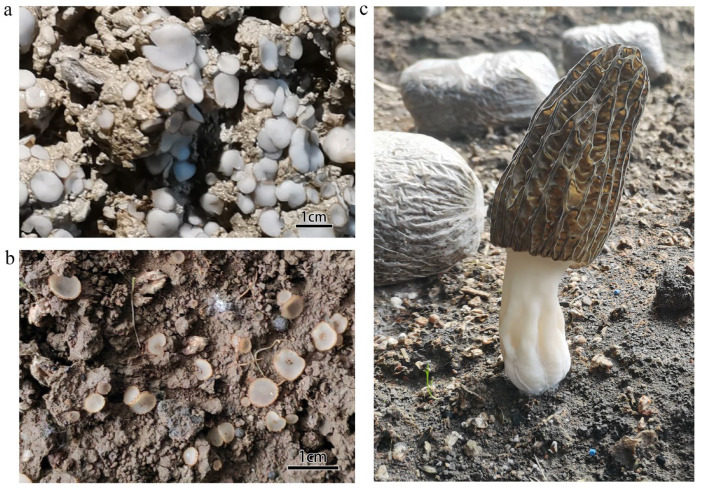
Soil samples collection. (**a**) Soil with large miscellaneous fungus growth (LF), (**b**) soil with small miscellaneous fungus growth (SF), (**c**) soil with normal morels.

The *Morchella* cultivation site was located in the greenhouse facilities of Baishengou Village, Ping’an District, Qinghai Province, situated on the Qinghai–Xizang Plateau. During the fruiting body formation stage, some of the greenhouses experienced significant fungal contamination of their soil, which hindered the growth of *Morchella* in the affected areas. Two distinct types of contaminating fungi were identified. The first contaminant (LF, Figure 1a) formed relatively large, disk-shaped fruiting bodies with diameters ranging from 8 to 20 mm. The second contaminant (SF, Figure 1b) produced smaller, funnel-shaped fruiting bodies with serrated edges, growing in clusters and ranging from 2 to 6 mm in diameter. Soil samples were collected from the rhizosphere of *Morchella* plants infected by LF (LF group), SF (SF group), and healthy, uninfected plants (CF; Figure 1c). Three random soil samples were collected for each type. Soil samples were collected at different locations and at different depths. Samples from each sampling site were taken from multiple small areas, and multiple soil samples collected at each sampling site were mixed to form a composite sample. Immediately after collection, the samples were flash-frozen in liquid nitrogen and stored at −80 °C for subsequent molecular and microbiological analysis.

### 2.2. Extraction, Sequencing, and Assembly of DNA from Soil Samples

The following describes our methodology: Accurately weigh a 0.1 g soil sample and extract the genomic DNA (gDNA) from the soil sample using a MagPure Soil DNA LQ Kit (Magen Biotech, Guangzhou, China). Subsequently, determine the concentration of the DNA using agarose gel electrophoresis and a NanoDrop 2000 (Kaiao Technology Development Co., Ltd., Beijing, China). Store the qualified gDNA at −20 °C for the construction of a metagenomic library.

To construct a metagenomic library, we employed the VAHTS™ Universal DNA Library Prep Kit for Illumina^®^ V3 (Vazyme Biotech Co., Ltd., Nanjing, China). Genomic DNA (gDNA) was sheared into random fragments using a Covaris S220 system (Gene Company Limited, Shanghai, China), followed by purification using a magnetic-bead-based method. The purified DNA fragments then underwent several essential steps, including 5′ phosphorylation, 3′ adenine tailing, adapter ligation, magnetic-bead-based purification, and PCR amplification, to complete the library preparation process. Sequencing was subsequently carried out by OE Biotech Co., Ltd., Shanghai, China.

Raw sequencing data were subjected to quality preprocessing using fastp (version 0.20.1) [36]. Following quality control, metagenomic assembly analysis was performed to enhance the recovery of genomic information for downstream functional annotation. The filtered and optimized sequences were assembled using MEGAHIT (version 1.2.9) [37,38,39,40,41], a De Bruijn graph-based assembler. In this process, De Bruijn graphs were constructed based on k-mer overlap relationships to generate contigs. Contigs longer than 500 bp were retained for further statistical analysis and subsequent functional characterization. MEGAHIT is a widely used tool for high-throughput metagenomic data assembly which is designed to efficiently process large-scale and complex metagenomic data. We chose MEGAHIT as the main assembly tool based on its high efficiency, accuracy, and compatibility in metagenomic data processing, which has been widely verified in the relevant literature.

### 2.3. Species Annotation

Open Reading Frames (ORFs) were predicted from the assembled contig sequences using Prodigal V2.6.3 [42] software, which were then translated into amino acid sequences. For the downstream processing of these ORF predictions, the MMSeqs2 V13.45111 software was employed to eliminate redundant sequences, resulting in a non-redundant initial gene set. In this context, non-redundant contiguous gene-encoded nucleic acid sequences were referred to as “genes”. Clustering was performed at an identity threshold of 95% and a coverage threshold of 90%, with the longest sequence within each cluster selected as the representative sequence.

Subsequently, the clean reads of each sample were aligned to this non-redundant gene set using Salmon V1.8.0. The abundances of each gene in the respective samples were quantified based on the number of aligned reads. Genes with fewer than two reads across all samples were filtered out to refine the final set of Unigenes. Gene abundance was further normalized by accounting for both the number of aligned reads and the gene length, providing a more accurate representation of gene expression levels across samples. From the resulting abundance table, which reflected gene counts across the samples, the number of genes present in each sample could be determined. To analyze the distribution of gene presence across multiple samples, random sampling was performed at varying sample sizes. This enabled the construction of dilution curves for Core and Pan genes, offering insights into gene overlap and variability across the samples.

Species annotation was performed using taxonomic information from the NR database, allowing for the identification and classification of species present in the sample. To quantify the abundance of each species, the total gene abundances corresponding to that species were summed. These species abundance values were then aggregated across multiple taxonomic levels, including domain, kingdom, phylum, class, order, family, genus, and species, to generate comprehensive abundance profiles at each respective level.

For alpha diversity analysis, diversity indices were computed based on the number of reads assigned to the species level. The statistical significances of the differences in alpha diversity across different experimental groups were then assessed.

### 2.4. Functional Annotation

DIAMOND V0.9.10.111 [43] software was used to align non-redundant gene sequences against various functional databases. Annotations were considered significant if the E-value was below 1 × 10^−5^ and, for each query, the alignment with the highest sequence similarity was selected to ensure the reliability of the functional annotation. In the second step, the alignment results were filtered to retain only those with the highest score for each sequence. Specifically, High-Scoring Pairs (HSPs) with bit scores greater than 60 were chosen for further analysis [44].

Based on these filtered alignment results, the relative abundance of genes at various functional levels was calculated. The relative abundance for each functional category was defined as the sum of the relative abundances of all the genes assigned to that category [45,46]. Functional annotation was performed using several databases, each with their own hierarchical structure. The KEGG database was categorized into six levels, the CAZy database into two levels, and the CARD into three levels. The gene abundance table for each sample was then constructed by counting the number of genes assigned to each functional level with a non-zero abundance.

Finally, using the abundance tables derived from these functional classifications, we conducted statistical analysis to evaluate the number of annotated genes at each functional level. Inter-group functional differences were assessed through statistical comparisons, followed by LEfSe analysis to identify biomarkers that could distinguish between the groups. This systematic approach ensured the comprehensive and robust functional profiling of the gene sequences across the samples.

### 2.5. Statistical Analyses

Hierarchical clustering analysis was employed to explore and compare the similarities and differences in gene abundance across multiple samples, typically represented through a dendrogram [47,48,49,50]. Initially, Bray–Curtis dissimilarity was used to calculate the pairwise distances between samples, yielding a beta diversity distance matrix. Hierarchical clustering was then performed on this matrix using the Unweighted Pair Group Method with Arithmetic Mean (UPGMA), which generated a tree-like structure that visually represented the relationships between the samples. This dendrogram served as a comprehensive tool for understanding the clustering patterns and similarities in gene abundance among the samples.

For differential gene expression analysis, the Kruskal–Wallis test was applied to assess differences in gene abundance across multiple groups. Post hoc pairwise comparisons were subsequently conducted on the top 10 genes exhibiting the largest effect sizes, enabling the more detailed investigation of groupwise differences.

### 2.6. Identification Methods for Pathogenic Fungi of Morchella

Test strains: Samples of large and small miscellaneous fungi were collected from the facility greenhouses where *Morchella* was cultivated in Ping’an District, Qinghai Province. The samples of large miscellaneous fungi were labeled as LF and the samples of small miscellaneous fungi were labeled as SF.

Medium: PDA medium (Solarbio Science & Technology Co., Ltd., Beijing, China).

Reagents: Ezup Fungal Genomic DNA Extraction Kit (Sangon Biotech, Shanghai, China), 2 × Taq PCR Master Mix, DNA primers, sterile water, etc.

The samples of LF and SF were first disinfected using 75% ethanol and subsequently rinsed with sterile water to remove any residual ethanol. After sterilization, the samples were meticulously cut into approximately 0.5 cm^3^ pieces using sterile scissors. Genomic DNA was then extracted from these samples using the Ezup Fungal Genomic DNA Extraction Kit, strictly following the manufacturer’s protocol to ensure high-quality DNA recovery. To amplify the internal transcribed spacer (ITS) region, PCR was performed using the primer pair of ITS1 (5′-TCCGTAGGTGAACCTGCGC-3′) and ITS4 (5′-TCCTCCGCTTATTGATATGC-3′). The resulting PCR products were subsequently sent to Sangon Biotech (Shanghai) Co., Ltd. for Sanger sequencing. The obtained sequencing data were analyzed using BLAST (https://blast.ncbi.nlm.nih.gov/Blast.cgi, accessed on 21 March 2024) against the NCBI database for species identification, enabling precise strain identification based on sequence homology.

## 3. Results

### 3.1. Quality Control and Statistical Analysis of Metagenomic Sequencing Data

After performing raw sequencing on the three groups of samples in this experiment, a total of 478,571,562 raw reads were obtained, totaling 71.8 GB in size, with an average of 7.98 GB per sample. Following quality control procedures, the data obtained are shown in Table S1, yielding a total of 478,107,602 clean reads, totaling 71.7 GB in size. The average GC content of the samples was 58.63%. The average Clean Q30 (1/1000 error rate) was 89.67%. Specifically, the CF group had an average of 55,370,205 clean reads, an average clean base of 8.28 GB, an average GC content of 61.18%, and an average Clean Q30 of 89.37%. The LF group had an average of 47,967,389 clean reads, an average clean base of 7.18 GB, an average GC content of 54.34%, and an average Clean Q30 of 90.50%. The SF group had an average of 58,183,754 clean reads, an average clean base of 8.38 GB, an average GC content of 60.37%, and an average Clean Q30 of 89.13%.

### 3.2. Metagenomic Sequencing Sequence Assembly and Preliminary Gene Prediction

The information on the contigs obtained from each sample and the results of gene prediction are shown in Table S2. A total of 6,969,514 contigs were obtained after assembly, with average contig lengths of 836,311 for the CF group, 549,926 for the LF group, and 936,934 for the SF group. Gene prediction was performed on the obtained contigs, resulting in a total of 6,018,104 Open Reading Frames (ORFs), with the average numbers of ORFs being 706,933 for the CF group, 502,775 for the LF group, and 796,326 for the SF group. Additionally, the average lengths of the contigs differed between groups, with 329.08 bp for the CF group, 513.73 bp for the LF group, and 356.18 bp for the SF group. Similarly, the average lengths of the predicted ORFs were 310.59 bp for the CF group, 374.45 bp for the LF group, and 330.16 bp for the SF group.

### 3.3. Species-Level Analysis of Each Sample

**Figure 2:**
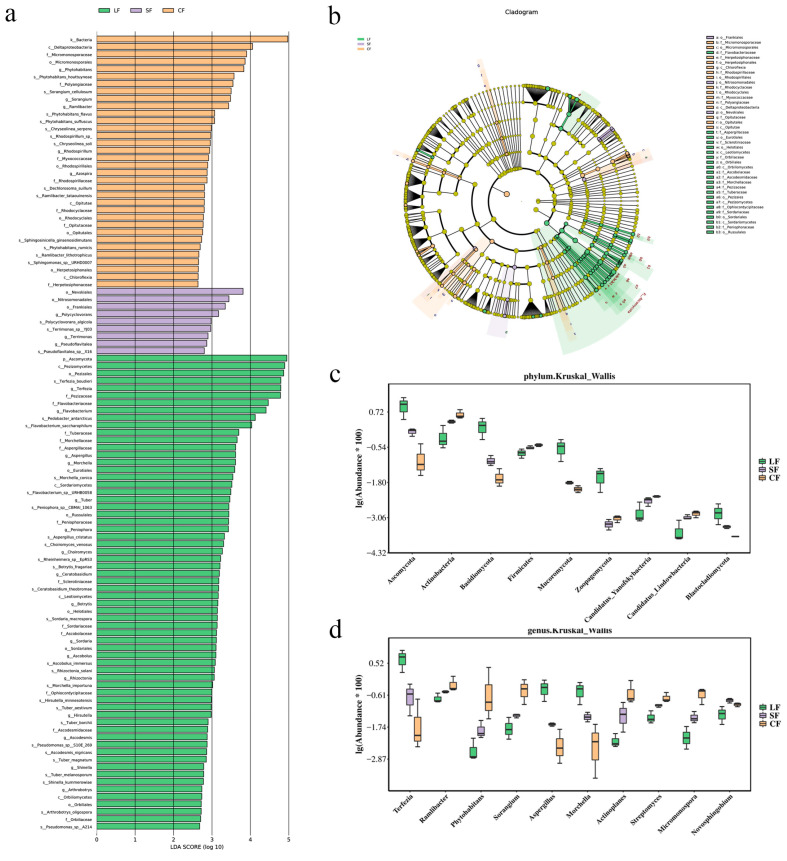
Distribution of polyphyletic species. (**a**) Differential species score map; (**b**) annotated branching diagram of different species; (**c**) boxplot of abundance information for top 10 species with significant differences at phylum level; (**d**) boxplot of abundance information for top 10 species with significant differences at genus level.

LEfSe analysis was conducted to identify microorganisms with significant differences between different sample site groups [51]. A total of 39 microbial biomarkers (LDA > 3) were identified, primarily belonging to the Actinobacteria, Bacteroidetes, and Proteobacteria phyla (Figure 2a,b). Among the characteristic microbial taxa associated with the CF group, one, namely the Bacteroidetes phylum, had an LDA value > 4. In contrast, among the characteristic microbial taxa associated with the LF group, 10 had LDA values > 4, with Ascomycota having the highest LDA value.

The abundance of species at different levels is shown in Figure S1. The bar chart in Figure 2c illustrates the species abundance at the phylum level across different sample groups. The dominant phyla with higher relative abundances in the CF group include Proteobacteria, Acidobacteria, and Bacteroidetes, with average relative abundances of 46%, 14%, and 9%, respectively. Similarly, in the SF group, Proteobacteria, Acidobacteria, and Bacteroidetes dominate, with relative abundances of 46%, 15%, and 12%, respectively. In the LF group, Proteobacteria, Bacteroidetes, and Ascomycota are the most abundant phyla, with average relative abundances of 44%, 18%, and 15%, respectively. Notably, the soil from the LF group, which is infected with *P. lohjaoensis*, exhibits a 6% increase in Bacteroidetes and a 14.8% increase in Ascomycota relative to the CF group, which contains healthy *Morchella*. In contrast, the soil from the SF group, infected with *T. gilva*, shows a modest increase of 0.8% in Ascomycota compared to the CF group.

The bar chart in Figure 2d presents the species abundance at the genus level for different samples, showing significant differences in the abundance and types of genus-level species between the CF, SF, and LF groups. In the CF group, the dominant genera with higher relative abundances include *Hydrogenophaga*, *Sphingomonas*, and *Cellvibrio*, with relative abundances of 5%, 4%, and 2%, respectively. In the SF group, the dominant genera are *Hydrogenophaga*, *Sphingomonas*, and *Polaromonas*, with relative abundances of 9%, 3%, and 2%, respectively. In contrast, the LF group is characterized by the higher relative abundances of *Pseudomonadaceae*, *Terfezia*, and *Pedobacter*, with average relative abundances of 8%, 9%, and 5%, respectively. Compared to the healthy *Morchella* CF group, the soil from the LF group infected with *P. lohjaoensis* has 7.1% more *Pseudomonadaceae*, 9% more *Terfezia*, 4.3% more *Flavobacterium*, and 0.5% more *Polaromonas*. In contrast, the soil from the SF group infected with *T*. *gilva* has 0.3% more *Terfezia* and 0.8% more *Massilia* compared to the healthy *Morchella* CF group. When comparing the LF group (infected with *P*. *lohjaoensis*) to the CF group (soil from healthy *Morchella*), the LF group exhibits a 7.1% increase in *Pseudomonadaceae*, a 9% increase in *Terfezia*, a 4.3% increase in *Flavobacterium*, and a 0.5% increase in *Polaromonas*. Conversely, the SF group, infected with *T*. *gilva*, shows only minor differences relative to the CF group, with a 0.3% increase in *Terfezia* and a 0.8% increase in *Massilia*.

### 3.4. Analysis of Alpha Diversity Indices for Soil Samples

The alpha and beta diversity indices of the soil samples are analyzed in Figures S2–S4. Based on the Shannon index [52] and Simpson index (Table S3), it can be seen that the SF group has the highest soil microbial community diversity, followed by the CF group, and the LF group has the lowest. Notably, while the CF group demonstrates relatively high microbial diversity, the LF group shows a marked reduction in diversity. This suggests that infection with *P. lohjaoensis* may diminish soil microbial diversity in *Morchella* habitats, while infection with *T. gilva* appears to enhance microbial diversity.

### 3.5. Analysis of Beta Diversity Indices for Soil Samples

#### 3.5.1. PCoA Analysis of Soil Samples

**Figure 3:**
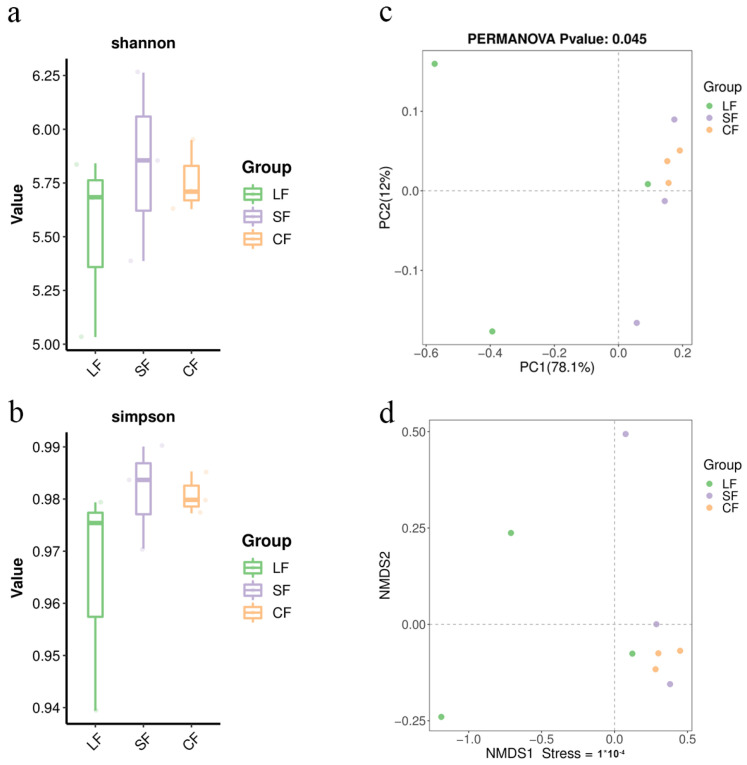
Analysis of soil samples. (**a**) Boxplot for comparing Shannon index between groups. (**b**) Boxplot for comparing Simpson index between groups. (**c**) PCoA analysis of soil samples (genus level). (**d**) NMDS analysis of soil samples (genus level).

Figure 3a,b shows the alpha box diagram, the results of the PCoA analysis for the soil samples are shown in Figure 3c. The soil microbial community of the LF group differs significantly from those of the CF and SF groups, while the difference between the soil microbial community of the SF group and the CF group is not significant.

Therefore, after infection with *P. lohjaoensis*, the species structure of the soil microbial community in which the diseased *Morchella* grows undergoes significant changes. However, after infection with *T*. *gilva*, there is no noticeable change in the microbial community structure of the soil in which the diseased *Morchella* grows.

#### 3.5.2. NMDS Analysis of Soil Samples

The green solid dots represent samples from the LF group, the purple solid dots represent samples from the SF group, and the yellow solid dots represent samples from the CF group. The samples were clustered using the NMDS method, as shown in Figure 3d. These results indicate that the LF group differs significantly from both the CF group and the SF group. With a stress value less than 0.05, the analysis provides a good representation. The difference between the SF group and the CF group is relatively small.

After infection with *P. lohjaoensis*, the species structure of the microbial community in the soil where the diseased *Morchella* grows undergoes significant changes. However, after infection with *T. gilva*, the microbial community structure in the soil where the diseased *Morchella* grows undergoes minimal changes.

### 3.6. Results of Non-Redundant Gene Functional Annotation

#### 3.6.1. Statistics of Non-Redundant Gene Functional Annotation Results

Using several databases, including the Kyoto Encyclopedia of Genes and Genomes (KEGG) [37,38], the Carbohydrate-Active Enzymes Database (CAZy) [39], and the Comprehensive Antibiotic Resistance Database (CARD) [53], we obtained non-redundant gene annotations for the soil microbial communities. Comparison with the KEGG database resulted in 1,279,305 non-redundant gene annotations, representing 47.93% of the total annotations. In contrast, comparison with the CAZy database yielded 34,597 non-redundant gene annotations, accounting for 1.3% of the total, while the CARD provided 4606 non-redundant gene annotations, which comprised 0.17% of the total.

#### 3.6.2. KEGG Analysis

**Figure 4:**
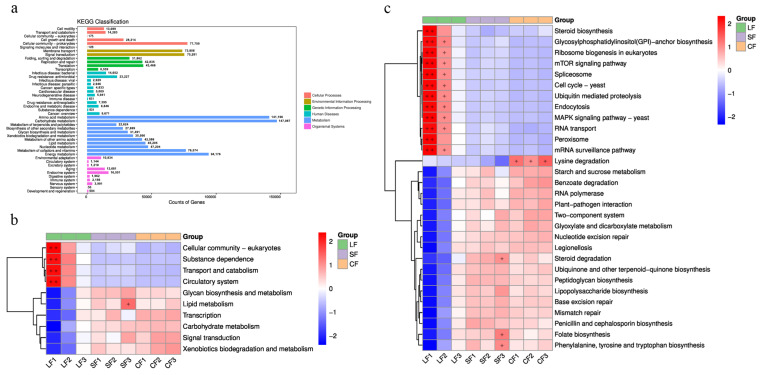
Functional difference analysis diagram of soil samples. (**a**) KEGG database annotated gene number statistical map; (**b**) differential functional heatmap of soil samples (KEGG level 2); (**c**) differential functional heatmap of soil samples (KEGG level 3). Note: star *p* < 0.05, double star *p* < 0.01. Same is true for figures below.

At level 1 of the KEGG database, a total of six categories of biological metabolic pathways were annotated (Figure 4a), including Cellular Processes (19.65%), Environmental Information Processing (21.07%), Genetic Information Processing (16.46%), Human Diseases (0.01%), Metabolism (22.45%), and Organismal Systems (20.36%).

Functional genes with significant differences in the KEGG level 2 hierarchy were plotted in a heatmap, as shown in Figure 4b. From the heatmap, it can be observed that the LF group differs significantly from both the SF group and the CF group. The functional genes with higher abundances include those related to the cellular community—eukaryotes, substance dependence, transport and catabolism, and the circulatory system. In contrast, no significant differences in functional gene composition were observed between the SF and CF groups. In these two groups, functional genes related to xenobiotic biodegradation and metabolism, signal transduction, carbohydrate metabolism, transcription, and lipid metabolism show higher abundance.

The top 30 pieces of abundance information with significant differences at the KEGG level 3 hierarchy were plotted in a heatmap, as shown in Figure 4c. The heatmap reveals notable differences between the LF group and both the SF and CF groups. In the LF group, the gene categories with higher abundance include steroid biosynthesis, Glycosylphosphatidylinositol (GPI)-anchor biosynthesis, ribosome biogenesis in eukaryotes, the mTOR signaling pathway, the Spliceosome, cell cycle yeast, Ubiquitin-mediated proteolysis, Endocytosis, MAPK signaling pathway yeast, RNA transport, Peroxisomes, and the mRNA surveillance pathway. In contrast, the CF group exhibits higher abundances of functional gene categories such as Lysine degradation, starch and sucrose metabolism, Benzoate degradation, and RNA polymerase. The SF group shows higher abundances of genes involved in folate biosynthesis, Phenylalanine, tyrosine, and tryptophan biosynthesis, lipopolysaccharide biosynthesis, and steroid degradation. Compared to the LF group and the SF group, the CF group has more genes related to the Lysine degradation pathway.

#### 3.6.3. CAZY Analysis

**Figure 5:**
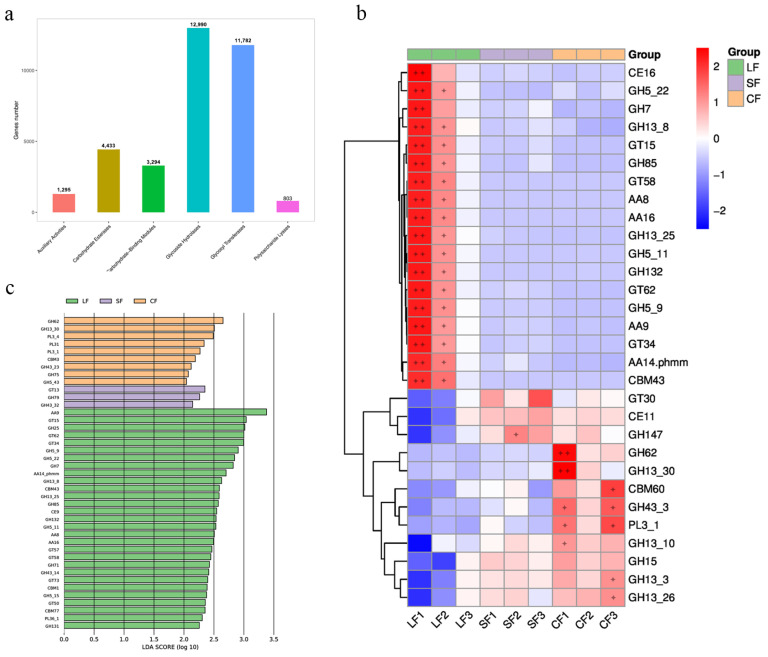
CAZY analysis chart. (**a**) Statistical map of the number of annotated genes in CAZY database; (**b**) heatmap map of gene differential function annotated by CAZY database; (**c**) CAZy database LEfSe analysis atlas.

The CAZY database is used to study carbohydrate-active enzymes, which include six categories: glycoside hydrolases (GHs), glycosyltransferases (GTs), Polysaccharide Lyases (PLs), Carbohydrate Esterases (CEs), Auxiliary Activities (AAs), and Carbohydrate Binding Modules (CBMs). Figure 5a presents the statistics of gene annotations using the CAZY database in this paper. According to the CAZY database, there are 1295 genes related to AAs, 4433 genes related to CEs, 3294 genes related to CBMs, 12,990 genes related to GHs, 11,782 genes related to GTs, and 803 genes related to PLs in our samples.

A significance test was conducted on all the annotated genes within the samples, and data with *p*-values less than 0.05 were selected. These data were then used to create a heatmap depicting the abundance information of the top 30 functionally significant genes, as shown in Figure 5b. In the LF group, the genes with higher abundances include one CE gene (CE16), eight GH genes (GH5_22, GH7, CH13_8, GH85, GH13_25, GH5_11, GH132, GH5_9), four GT genes (GT15, GT58, GT62, GT34), four AA genes (AA8, AA16, AA9, AA14.phmm), and one CBM gene (CBM43). Only one additional glycosyltransferase was identified in the SF group (Figure 5c). Here, the genes with higher abundance included one glycosyltransferase (GT30), one carbohydrate esterase (CE11), and three glycoside hydrolases (GH147, GH15, GH13_3). In the CF group, the higher-abundance genes included seven GH genes (GH62, GH13_30, GH43_3, GH13_10, GH15, GH13_3, GH13_26), one CBM gene (CBM60), and one PL gene (PL3_1). Notably, the SF group exhibited a significantly lower diversity of GHs compared to both the LF and CF groups. This differential distribution suggests distinct functional profiles across the groups, highlighting potential variations in carbohydrate degradation and utilization pathways.

#### 3.6.4. CARD Analysis

**Figure 6:**
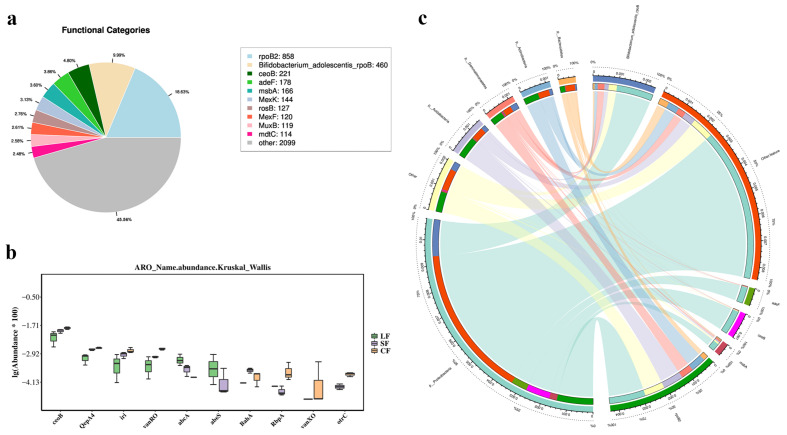
CARD analysis chart. (**a**) CARD annotation top 10 ARO chart; (**b**) CARD annotation top 10 ARO significant differences boxplot diagram; (**c**) Circos diagram.

A total of 4582 resistance genes were annotated in the CARD resistance gene database, with rpoB2 (858, 18.60%) and Bifidobacterium adolescentis rpoB (460, 9.99%) identified as rifamycin resistance genes (Figure 6a). Other relatively abundant resistance genes included MexK, ceoB, adeF, msbA, rosB, MexF, MexB, and mdtC. The Kruskal–Wallis test revealed significant differences in resistance functions among the three soil microbiota groups (Figure 6b). The CF group exhibited higher abundances in resistance functions related to antibiotic target modification, target replacement, efflux, target protection, and inactivation. The LF group was notably enriched in functions associated with reduced antibiotic permeability, which was significantly higher than in the CF and SF groups, with other resistance functions being almost absent. The CF group also showed higher abundances in resistance functions related to antibiotic target modification, target protection, and inactivation.

The Circos analysis (Figure 6c) revealed that Proteobacteria played a prominent role in resistance gene functions, accounting for a large proportion of the total resistance in the circular plot. The Acidobacteria phylum primarily harbored two resistance genes: rpoB2 (858, 18.60%) and Bifidobacterium adolescentis rpoB (460, 9.99%).

### 3.7. Molecular Biological Identification of Pathogenic Fungi

Samples of large (LF) and small (SF) pathogenic fungi with significant morphological differences were collected. After amplification using rDNA-ITS primers, the LF pathogenic fungi yielded a 592 bp fragment. BLAST comparison with the rRNA/ITS database sub-library of the NCBI database showed a 98% similarity to the sequence with accession number NR_148063, and it was preliminarily identified as a fungus belonging to the genus *Peziza*, specifically *Peziza lohjaoensis*. The SF pathogenic fungi, after amplification, yielded a 529 bp fragment. BLAST comparison with the rRNA/ITS database sub-library of the NCBI database showed the highest similarity of 96% to the sequence with GenBank accession number NR_160170.1, and it was preliminarily identified as *Tricharina gilva*.

## 4. Discussion

### 4.1. Impact of Fungal Infection on the Soil Microbial Community of Morchella sextelata

During the cultivation of *Morchella*, farmers often utilize soil from consecutive cropping cycles for covering, suggesting that these soils harbor beneficial microbial communities that may enhance the growth of *Morchella* [54,55]. Research by Ower has identified a beneficial mold, *Costantinella cristata* Matruchot, which promotes *Morchella* growth under controlled indoor conditions [10]. Additionally, in a study by Zhang et al., it was found that *Actinomycetes* in the soil produce antibiotics, enzymes, and enzyme inhibitors which promote the growth of *Morchella*, suggesting that *Actinomycetes* may be the dominant phylum of soil microorganisms in *Morchella* cultivation [56]. In this study, at the phylum level, the dominant microbial phyla in healthy *Morchella* were Proteobacteria, Acidobacteria, Bacteroidetes, and Actinobacteria. This conclusion is consistent with the research of Kang et al. [57], Liu et al. [8], and Zhang et al. [58], among others. These phyla are also major components of the soil microbial community in the natural habitat of *Morchella rufobrunnea* [59] and in semi-synthetic substrates for *Morchella* cultivation [60], indicating that these microorganisms play an important role in the life cycle of *Morchella*. Furthermore, this study found that the number of Ascomycota increased and the number of Acidobacteria decreased in soil where *P. lohjaoensis* was growing. This suggests that the variety of *Morchella* infected with *P. lohjaoensis* may be a fungus belonging to Ascomycota, or that a fungus within Ascomycota may make *Morchella* susceptible to *P. lohjaoensis*.

Studies have shown that in areas with medium to high yields of cultivated *Morchella* there are numerous unclassified fungi belonging to the Discomycetes class. At the bacterial phylum level, Bacteroidetes account for a relatively high proportion, while at the fungal phylum level, Ascomycota account for a very high proportion [61]. In this study, according to the Shannon and Simpson indices of soil alpha diversity, after infection with *P. lohjaoensis*, the diversity of the soil microbial community in *Morchella* decreased, while after infection with *T. gilva*, the diversity of the soil microbial community in *Morchella* increased. This may indicate that *P. lohjaoensis* has the ability to inhibit the growth and reproduction of other microorganisms. The ability of Discomycetes fungi to inhibit microbial growth may be one of the reasons for the high yields of cultivated *Morchella*. At the taxonomic phylum level, the soil in the LF group with *P. lohjaoensis* growth had a 6% higher relative abundance of Bacteroidetes and a 14.8% higher relative abundance of Ascomycota compared to the healthy *Morchella* CF group. The SF group with *T. gilva* growth had a 0.8% higher relative abundance of Ascomycota compared to the healthy *Morchella* CF group. This indicates that Bacteroidetes and Ascomycota are dominant phyla in the soil of cultivated *Morchella*, which is consistent with previous research results [61].

Benucci et al. found in their study of the microbial communities in the soil and fruiting bodies of cultivated *Morchella* that bacteria of the genus *Pseudomonadaceae* can promote the growth of *Morchella* [62]. In this study, at the genus taxonomic level, the dominant bacterial genera in the soil of the LF group with *P. lohjaoensis* growth were *Pseudomonadaceae* and *Flavobacterium*. This finding offers valuable insights into the potential role of *P. lohjaoensis* infection in modulating microbial communities within the soil and its subsequent impact on the growth dynamics of *Morchella*.

### 4.2. Impact of Fungal Infection on Soil Functional Genes in Morchella sextelata

Common diseases affecting edible mushrooms include *Trichoderma*, *Penicillium*, and *Fusarium rubrum*, among others [63,64,65]. Studies have found that *Diploöspora longispora* can cause fungal wilt disease in edible mushrooms [66]. In a study by Tong et al., drought stress on *Morchella* was shown to upregulate genes involved in starch and sucrose metabolism, arginine and proline metabolism, and ammonium sulfate metabolism, suggesting that these genes play a role in facilitating the growth and stress tolerance of *Morchella* [67]. In this study, the KEGG level 2 heatmap showed that the CF group (healthy *Morchella*) and the SF group (with *T. gilva* growth) had abundant genes related to biological metabolism and carbohydrate metabolism; this may be conducive to the microbial growth and diversity of SF group. The LF group (with *P. lohjaoensis* growth) had higher abundances of functional genes related to eukaryotic community-associated genes, substance dependence-related genes, substance transport and catabolism-related genes, and circulatory system-related genes. According to the heatmap of the top 30 most abundant genes with significant differences at KEGG level 3, the CF group had high abundances of genes related to starch and sucrose metabolism, and only the CF group had a high abundance of genes related to the Lysine degradation pathway. Therefore, it can be concluded that infection with *T. gilva* and *P. lohjaoensis* affects the Lysine degradation ability of *Morchella*, and it may also affect the survival of microorganisms in healthy soil. The above two studies can corroborate each other, and subsequent research can further distinguish the specific degree of impact of the two.

The growth and development of edible mushrooms depend on a variety of nutrients, including carbon sources, nitrogen sources, vitamins, inorganic salts, and trace elements [68]. Research by Wang et al. indicates that different strains of *Morchella* exhibit variations in their metabolic utilization of carbon sources [69]. In particular, Wei et al. pointed out in their study that lipids may serve as the primary carbon source during the growth of *Morchella sextelata* [70]. In this study, the LF group with *P. lohjaoensis* infection showed a reduced abundance of genes associated with lipid metabolism, yet a higher abundance of genes related to steroid biosynthesis. In contrast, the healthy CF group and the SF group with *T. gilva* infection exhibited a higher representation of genes involved in lipopolysaccharide biosynthesis. These findings suggest that *P. lohjaoensis* infection may impair the ability of *Morchella* to utilize lipids in the soil, potentially inhibiting its growth. Interestingly, the SF group infected with *T. gilva* displayed an elevated abundance of genes associated with folate biosynthesis. This observation implies that *T. gilva* infection could enhance the utilization of vitamins, such as folate, in the soil, which may, in turn, support the growth and development of *Morchella* and microorganisms.

### 4.3. Impact of Fungal Infection on Soil Microbial Carbohydrate-Enzyme-Related Genes in Morchella sextelata

The organic macromolecules such as cellulose and lignin, which are nutrients provided by nature to the mycelium, require the release of corresponding extracellular enzymes by the mycelium for their degradation and utilization. The expression of enzyme genes related to nutrition and metabolism in edible fungi is directly related to their nutrient utilization [71]. Carbohydrates in soil microorganisms play a crucial role in the growth and development of fungi. For example, studies have found that *Sphingomonas* is considered beneficial to mushroom growth, can proliferate during composting, and possesses strong lignocellulose degradation capabilities [72,73,74]. This study found that the genus *Sphingomonas* was relatively abundant in the *Morchella* CF group, potentially aiding in the mycelial elongation and nutritional metabolism of *Morchella*. In contrast, the LF group had only one abundant carbohydrate-active enzyme gene, resulting in the low utilization of carbohydrates in the soil, which may inhibit the growth of *Morchella*.

In Huang Peng’s study, glycoside hydrolases, key enzymes in the organic carbon cycle of soil, were shown to play an essential role in the degradation of glycosidic bonds in sugar-containing compounds. These enzymes assist microorganisms in breaking down polysaccharides, carbohydrate esters, and lignin, thereby facilitating microbial growth by providing essential carbon sources [75]. This study found that the diversity of glycoside hydrolases in the SF group infected with *T. gilva* was significantly lower compared to the healthy *Morchella* CF group. This suggests that infection by *T. gilva* may impair the ability of *Morchella* to utilize carbohydrates in the soil, thereby inhibiting its growth. In contrast, previous studies have demonstrated that glycoside hydrolases produced by *Setosphaeria turcica* play a crucial role in degrading the lignocellulosic structures of plant tissues, such as corn leaves, which aids in the fungal invasion of the host and the onset of disease symptoms [76]. This could explain why, despite a higher abundance and diversity of glycoside hydrolases in the LF group infected with *P. lohjaoensis*, the growth and development of *Morchella* were still compromised.

### 4.4. Effects of Fungal Infection on Resistance Gene Functions of Soil Microbiota in Morchella sextelata

In this study, a total of 4582 resistance genes were identified within the soil microbiota of *Morchella*, indicating the presence of diverse resistance mechanisms within the soil microbial community. These findings are consistent with previous studies [77,78]. Through Kruskal–Wallis testing, we observed significant differences in resistance functions across different soil groups (CF, LF, SF). This observation aligns with the results of Allen et al. [79], who noted significant functional differentiation in microbial resistance mechanisms under varying environmental conditions. In addition, our results also suggest that the high abundance of antibiotic outflow and target protection mechanisms in the CF group may be related to the selective pressure of antibiotics in the environment [80], while the reduced enrichment of antibiotic permeability in the LF group may reflect the adaptation strategies of the microorganisms in this group to specific environmental pressures. *Peziza lohjaoensis* may be able to withstand the stress of chemicals such as antibiotics to survive.

The Circos analysis in this study further revealed the dominant role of Proteobacteria in resistance gene functions, which is consistent with the findings of D’Costa et al. [81], who observed a widespread distribution of resistance genes across various environmental samples. Additionally, the rpoB2 and Bifidobacterium adolescentis rpoB genes within the Acidobacteria were found to play a significant role in resistance functions, suggesting that microorganisms from this phylum may possess unique adaptive strategies under specific antibiotic pressures [79]. These results underscore the phylum-specific contributions of different microbial taxa to resistance functions, providing important insights into the diversity of resistance mechanisms in *Morchella* soil microbiota.

### 4.5. Identification of Pathogenic Fungi in Morchella sextelata

Previous studies have indicated that cultivated *Morchella* soil can spontaneously harbor Pezizaceae fungi [81]. Zhang et al. observed an increased abundance of unclassified fungi within the class Pezizomycetes in *Morchella* cultivation areas [61]. In the present study, we employed both morphological characterization and rDNA-ITS sequencing to identify fungal pathogens associated with *Morchella* cultivation in Qinghai Province. The results revealed that the pathogen from the LF group exhibited 98% sequence similarity to the reference sequence (accession number NR_148063) and was preliminarily identified as *Peziza lohjaoensis*. This finding contributes to the species identification and resource distribution of this fungal genus. For the SF group, the pathogen showed the highest sequence similarity (96%) to the sequence with accession number NR_160170.1 and was tentatively identified as *Tricharina gilva*, a species within the genus *Tricharina* of the Pyronemataceae family in the order Pezizales. This morphological identification aligns with previous reports by Zhuang et al. [82]. Notably, *T. gilva* has been recorded in various ecosystems, including saline–alkali soils in Inner Mongolia [83], sandy habitats with *Ulmus pumila* [84], and in the Qilian Mountains, where *Picea crassifolia* grows [85]. Additionally, *T. gilva* is known as an endophytic fungus of the medicinal plant *Warburgia ugandensis*, capable of synthesizing bioactive compounds such as flavonoids, tannins, and alkaloids, which have beneficial effects on plant health [86]. These observations underscore the functional diversity of this fungus across different ecological contexts. In future studies, we aim to investigate the infection pathways and mechanisms by which these pathogens affect *Morchella*. Furthermore, we will explore effective antimicrobial strategies to mitigate pathogen-induced yield losses, ultimately improving *Morchella* production and reducing economic impact in cultivation areas.

Overall, studying the impact of *Morchella sextelata* fungal pathogen infection on soil microbial communities helps us understand how diseases alter soil microbial composition and function, affecting crop health and growth. Currently, the focus of Stropharia rugosoannulata cultivation is on prevention, especially preventing fungal infections. Key to this is selecting high-quality strains with a pure, dense mycelium, high vitality, strong disease resistance, and no contamination. Prior to inoculation, the thorough sterilization of both the strain and soil is essential. If contamination occurs during cultivation, infected mushroom bags should be destroyed and the environment should be disinfected again. Temperature and humidity adjustments within optimal ranges can help suppress high-temperature, high-humidity pathogens. Although chemical fungicides are cost-effective, they may leave toxic residues, making biocontrol a more favorable option. Future research will focus on screening beneficial fungi that can antagonize fungal diseases. Microbial communities play a critical role in soil ecosystems, promoting plant health and inhibiting pathogens. If specific microbial communities decline or become imbalanced in diseased soils while remaining stable in healthy soils, these microbes may be key to biocontrol strategies. By studying microbial changes in relation to diseases, we can develop microbe-driven biocontrol methods to enhance crop resistance, improve soil health, and support sustainable agriculture, contributing to eco-friendly, sustainable farming systems.

## 5. Conclusions

This study utilized metagenomic sequencing technology to analyze the impact of fungal contamination in the soil of *Morchella* cultivation sheds in Ping’an District, Qinghai Province, on the microbial community of *Morchella* mushrooms. Nine soil samples from three treatments were analyzed. After the quality control of the data, species-level and functional-gene-level analyses were conducted using methods such as the alpha diversity index, the KEGG database, the CAZY database, and the CARD. The following conclusions were drawn:

1. Infection by *P. lohjaoensis* resulted in a lower microbial diversity in the *Morchella* soil community compared to the control group, while infection by *T. gilva* led to a higher microbial diversity compared to the control group.
2. Both the *P. lohjaoensis* and *T. gilva* infections altered the microbial community in the *Morchella* soil, with differences in the dominant phyla and genera observed in the different soil samples. After infection with *P. lohjaoensis*, the dominant phyla with relatively higher abundances included Proteobacteria (44%), Bacteroidetes (18%), and Ascomycota (15%). The dominant genera with relatively higher abundances were *Pseudomonadaceae* (8%), *Terfezia* (9%), and *Pedobacter* (5%). After infection with *T. gilva*, the dominant phyla with relatively higher abundances included Proteobacteria (46%), Acidobacteria (15%), and Bacteroidetes (12%). The dominant genera with relatively higher abundances were *Hydrogenophaga* (29%), *Sphingomonas* (3%), and *Polaromonas* (2%).
3. After infection by *P. lohjaoensis*, the microbial community structure in the soil where *Morchella* was growing underwent significant changes. However, after infection by *T. gilva*, the microbial community structure in the *Morchella* growth soil did not differ significantly from that of the healthy control group.
4. Fungal infection significantly altered microbial resistance functions in the soil where *Morchella* mushrooms were grown. Following infection by *P. lohjaoensis*, resistance functions were enriched in antibiotic permeability reduction. In contrast, infection by *T. gilva* led to an enrichment of resistance functions related to antibiotic target modification, target protection, and inactivation.
5. Using rDNA-ITS for strain identification, the pathogen in the LF group was identified as *P. lohjaoensis*, belonging to the order Pezizales, family Pezizaceae, and genus *Peziza*. The pathogen in the SF group was identified as *T. gilva*, belonging to the order Pezizales, family Pyronemataceae, and genus *Tricharina*.
6. Each sample in this study was replicated only three times, which may not be sufficiently representative of the overall sample. Therefore, the results may be limited by sample size and there is some statistical uncertainty. Future studies should consider increasing the sample size to improve the reliability and representativeness of the results.

## Data Availability

The supported data are contained within the Supplementary Materials, Tables S1–S3.

## Supplementary Materials

The following supporting information can be downloaded at https://www.mdpi.com/article/10.3390/jof11040264/s1. Table S1. Quality control of post-metagenomic gene data. Table S2. Metagenomic sequencing technology sequence assembly after contigs information. Table S3. The alpha diversity indexes of the samples were analyzed at the species level. Figure S1. Species abundance at different levels: a. Distribution of polyphyletic species (phylum level), b. distribution of polyphyletic species (class level), c. distribution of polyphyletic species (order level), d. distribution of polyphyletic species (family level), e. distribution of polyphyletic species (genus level), f. distribution of polyphyletic species (species level). Figure S2. A PCA diagram. a–f are the PCA analysis charts of class, family, genus, order, phylum, and species at the taxonomic level. The more similar the species composition, the closer the distance reflected in the PCA map. Figure S3. A PCoA diagram. a–f are the PCoA analysis charts of species, phylum, order, genus, class, and family at the taxonomic level. Each point in the figure represents a sample, the same color indicates the same group. The closer the samples in the same group are, and whether there is a significant distance between them and other groups, indicates that the biological duplication within the group is good. Figure S4. An NMD diagram. a–f are the NMDS analysis charts of class, family, genus, order, phylum, and species at the taxonomic level. Each point in the figure represents a sample, the same color indicates the same group. The closer the samples in the same group are, and whether there is a significant distance between them and other groups, indicates that the biological duplication within the group is good.
