# Supplementary material for: The Effects of Fungal Pathogen Infestation on Soil Microbial Communities for Morchella sextelata Cultivation on the Qinghai–Xizang Plateau
Source: J Fungi (Basel). 2025 Mar 28;11(4):264. doi: 10.3390/jof11040264 (PMC12029088; doi:10.3390/jof11040264)
Supplement: Supplementary file 1 [file jof-11-00264-s001.zip › Supplementary materials.pdf]

Table S1. Quality control of post-metagenomic gene data

Samples	Data					
	Raw reads（bp）	Clean reads（bp）	Raw base	Clean base	GC（%）	Q30（%）
CF1	55795646	55743592	8.37G	8.34G	60.87%	89.42%
CF2	55886578	55835000	8.38G	8.35G	61.31%	89.11%
CF3	54582676	54532024	8.19G	8.15G	61.37%	89.57%
LF1	42591278	42539682	6.39G	6.37G	50.43%	91.64%
LF2	46909568	46855582	7.04G	7.02G	52.50%	90.87%
LF3	54555430	54506902	8.18G	8.15G	60.10%	88.98%
SF1	58235760	58183754	8.74G	8.70G	60.43%	89.37%
SF2	53636680	53585092	8.05G	8.01G	60.80%	89.02%
SF3	56377946	56325974	8.46G	8.42G	59.88%	89.01%

Table S2. Metagenomic sequencing technology sequence assembly after Contigs information statistical table

**Samples**	**Data**			
	Contigs number	ORFs number	Mean length of Contigs（bp）	Mean length of ORFs（bp）
CF	836311	706933	329.08	310.59
LF	549926	502775	513.73	374.45
SF	936934	796326	356.18	330.16

Table S3. The alpha diversity index of samples was analyzed at the species level

Group	CF	LF	SF
Shannon	5.7625±0.16670451	5.5195±0.428201401	5.8351±0.438513281
Simpson	0.9808±0.004088387	0.9647±0.022030282	0.9814±0.009978882

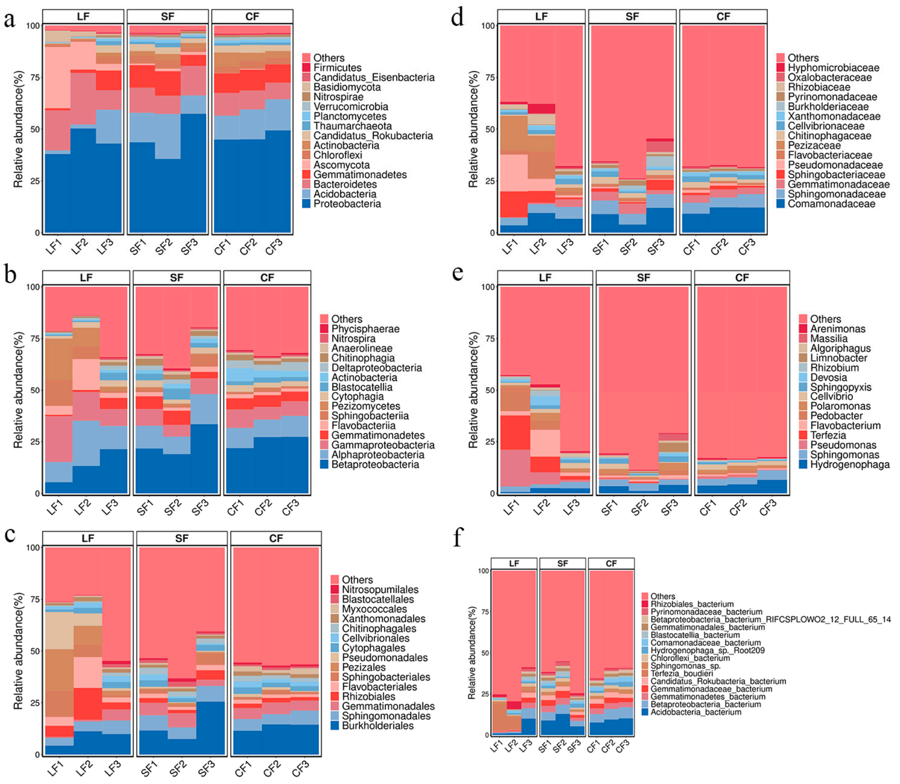

Figure S1. Species abundance at different levels. **a.** Distribution of polyphyletic species (phylum level), **b.** Distribution of polyphyletic species (class level), **c.** Distribution of polyphyletic species (order level), **d.** Distribution of polyphyletic species (family level), **e.** Distribution of polyphyletic species (genus level), **f.** Distribution of polyphyletic species (species level).

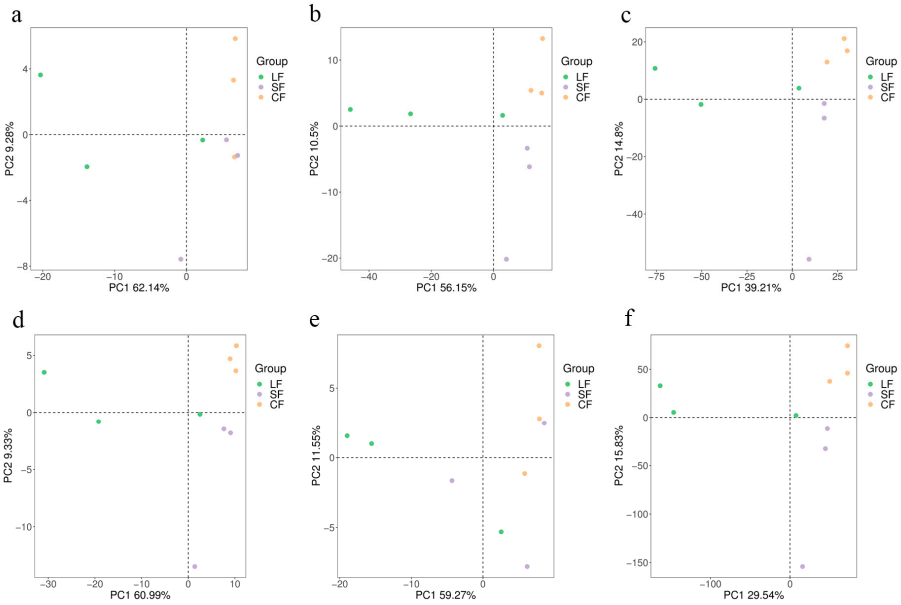

Figure S2. PCA diagram. **a** to **f** are the PCA analysis charts of class, family, genus, order, phylum and species at the taxonomic level. The more similar the species composition, the closer the distance reflected in the PCA map.

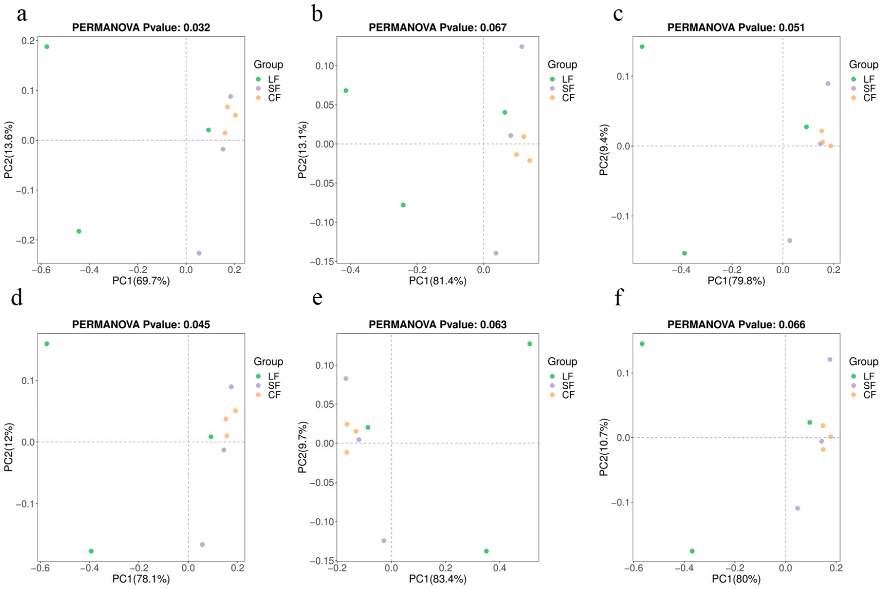

Figure S3. PCoA diagram. **a** to **f** are the PCoA analysis charts of species, phylum, order, genus, class and family at the taxonomic level. Each point in the figure represents a sample, the same color is the same group, the closer the samples in the same group are, and there is a significant distance between them and other groups, indicating that the biological duplication within the group is good.

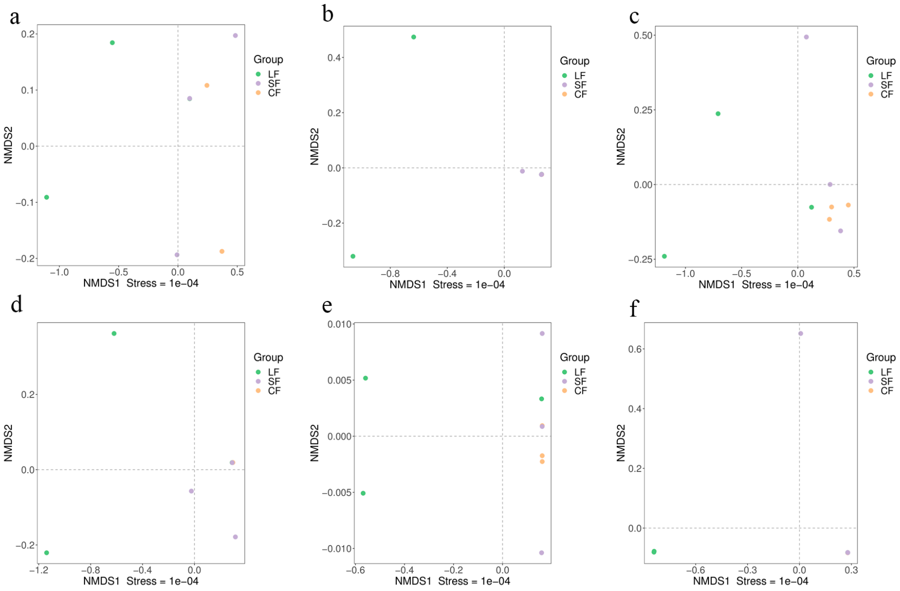

Figure S4. NMD diagram. **a** to **f** are the NMDS analysis charts of class, family, genus, order, phylum and species at the taxonomic level. Each point in the figure represents a sample, the same color is the same group, the closer the samples in the same group are, and there is a significant distance between them and other groups, indicating that the biological duplication within the group is good.
